# 

**Published:** 1917-05

**Authors:** 


Vol. XXXII MAY, 1917 No. 11

### TEXAS Medical Journal

